# Endothelial Dysfunction in Systemic Sclerosis: The Passive Leg Movement Technique in the Assessment of Nitric Oxide–Mediated Vascular Impairment

**DOI:** 10.1002/acr2.90017

**Published:** 2026-03-30

**Authors:** Silvia Guella, Giovanni Baldassarre, Alessandro Gratton, Lucrezia Zuccarelli, Elena Treppo, Emma Di Poi, Bruno Grassi, Luca Quartuccio

**Affiliations:** ^1^ Rheumatology Division, Academic Hospital “Santa Maria della Misericordia” Udine Italy; ^2^ CMID‐Nephrology and Dialysis Unit, San Giovanni Bosco Hub Hospital, Department of Clinical and Biological Sciences University of Turin Turin Italy; ^3^ Department of Medicine University of Udine Udine Italy

## Abstract

**Objective:**

To evaluate the impairment of nitric oxide (NO)–mediated vascular function in patients with systemic sclerosis (SSc) compared with healthy controls through passive leg movement (PLM), which is a noninvasive technique largely dependent on NO bioavailability.

**Methods:**

Twenty‐one patients with SSc and 21 age‐ and sex‐matched healthy controls were recruited. PLM was performed in a sitting position, and blood flow was assessed at the level of the right common femoral artery, distal to the inguinal ligament, and approximately 2 cm from the femoral artery bifurcation, by measuring flow velocity and vessel diameter. Resting blood flow, peak flow during PLM, and the area under the blood flow versus time curve (AUC) were calculated. Patients with SSc were stratified by disease phenotype and treatment regimens.

**Results:**

Patients with SSc showed significantly reduced PLM responses compared with controls (peak flow: 467.7 ± 97.8 vs 552.5 ± 151.9, *P* = 0.038; ΔPeak:153.3 ± 59.8 vs 220.2 ± 82.7, *P* = 0.005; AUC: 57.1 ± 38.9 vs 89.3 ± 38.1, *P* = 0.01), indicating impaired NO‐mediated vasodilation. No significant differences in PLM results were found across SSc phenotypes. An inverse correlation was observed between modified Rodnan Skin Score and femoral artery diameter (*P* = 0.001), suggesting vascular remodeling. Pharmacologic treatments, including vasoactive and immunosuppressive agents, did not significantly impact PLM parameters. Statin use was associated with lower baseline blood flow (*P* = 0.031).

**Conclusion:**

PLM responses are significantly reduced in patients with SSc, confirming the role of NO‐mediated endothelial dysfunction in SSc, independent of disease subtype or treatment status. PLM may serve as a noninvasive biomarker for microvascular health in SSc and underscore the need for therapies that directly target the endothelium. Larger, longitudinal studies are warranted to confirm these results and explore PLM's prognostic utility.

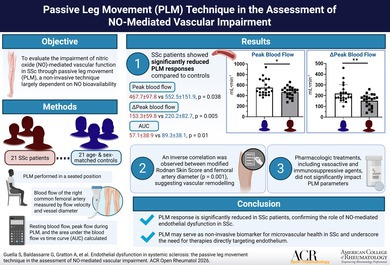

## INTRODUCTION

Systemic sclerosis (SSc) is a rare autoimmune connective tissue disease characterized by vasculopathy and progressive fibrosis, affecting the skin, lungs, heart, kidneys, and gastrointestinal tract.^1^ The pathogenesis of SSc involves immune dysregulation, abnormal fibroblast activity, and early, crucial endothelial dysfunction, which contributes to both early signs (eg, Raynaud phenomenon) and severe complications like pulmonary arterial hypertension (PAH).^1^, ^2^ Endothelial dysfunction in SSc includes up‐regulation of adhesion molecules, an imbalance between vasodilators (eg, nitric oxide [NO] and prostacyclin) and vasoconstrictors (eg, endothelin) mediators, and mesenchymal transformation, all contributing to tissue damage and impaired vascular repair.^2^ DAMPs, cytokines, and reactive oxygen species, along with growth factors such as platelet‐derived growth factor and vascular endothelial growth factor (VEGF), amplify inflammation and promote vascular remodeling.^2^, ^3^, ^4^ Anti–endothelial cell antibodies, found in 22% to 68% of patients with SSc, further impair vascular repair by inducing endothelial cell apoptosis and reducing endothelial progenitor cells.^5^, ^6^ Immune dysregulation also plays a role in promoting a proinflammatory and profibrotic environment.^7^

To assess endothelial dysfunction and vascular responses in terms of structure and function, noninvasive techniques like flow‐mediated dilatation (FMD) and passive leg movement (PLM) are used.^8^, ^9^ FMD, through Doppler ultrasound, evaluates changes in artery diameter induced by hyperemia following temporary occlusion of the brachial artery caused by inflation of a blood pressure cuff.^8^ PLM assesses peripheral vascular function through the passive movement of the leg. The increase in blood flow (hyperemia), caused by a 90° range of movement, is measured by Doppler ultrasound, and it was demonstrated to be a simple and reliable test to evaluate peripheral vascular function, with up to 80% of PLM‐induced hyperemia being NO‐mediated.^9^ Both techniques have been successfully applied across various populations, including patients with rheumatic diseases.^10^, ^11^, ^12^ Particularly, PLM was employed to study young and elderly patients, active and sedentary, as well as patients with chronic inflammatory diseases.^12^, ^13^, ^14^, ^15^ However, only one study has evaluated PLM response in patients with SSc, showing reduced hyperemia compared to healthy controls.^12^ Furthermore, to date, there is no analysis about PLM response in patients with SSc based on disease phenotype and treatments employed.

This study aims to evaluate NO‐mediated vascular impairment in patients with SSc using PLM and to explore correlations with disease phenotype, severity, and the effects of immunosuppressive or vasoactive therapies.

## MATERIALS AND METHODS

### Ethical approval

The study protocol was approved by the University of Udine Department of Medicine Institutional Review Board (227/2024) and involves two research units: the Laboratory of Exercise Physiology and the Rheumatology Division of Udine. All participants provided written informed consent before participating in the experiments.

### Participants

A total of 21 patients with SSc and 21 healthy, age‐ and sex‐matched controls were recruited. All patients met the classification criteria for a diagnosis of SSc,^16^ and were divided into three subgroups, according to the phenotype and severity of the disease: limited cutaneous SSc (lcSSc), lcSSc with PAH, and diffuse cutaneous SSc (dcSSc). Clinical, laboratory, and instrument data were retrospectively collected from the patients’ medical records.

### Experimental protocol

All participants visited the laboratory once, and the experimental session lasted approximately one hour. Participants arrived at the laboratory in the afternoon, at least 2 hours postprandial, and were instructed to refrain from exercise, alcohol, and caffeine for 24 hours before the assessment. The environmental conditions of the room remained stable throughout the session. Oral vasoactive therapies were continued, whereas for patients receiving intravenous prostacyclin, PLM assessment was performed after a mean interval of 11 ± 6 days from the last infusion. During the visit, a whole‐body bioimpedance analysis (BIA) was performed by a phase‐sensitive single‐frequency device (BIA 101 BIVA, AKERN srl), followed by a PLM experimental session (see details below).

### PLM test

Patients remained at rest for at least 10 minutes before the test, in a sitting position. Blood flow was assessed by measuring blood flow velocity and vessel diameter in the common femoral artery (CFA), distal to the inguinal ligament, and approximately 2 cm from the femoral artery bifurcation. An ultrasound system with a linear array transducer was used, and two‐dimensional measurements of the arterial lumen were made from B‐mode images in longitudinal view. Vessel diameter was measured at the same point in the cardiac cycle (peak of the R wave derived from the integrated electrocardiogram [EKG] system). A trained investigator conducted baseline measures for 30 s and then blood flow measurements were performed during one minute of PLM of the right leg. The movements were performed across a 90° range of motion (180° to 90° to 180°) at 1 Hz, following a metronome. Patients were instructed to remain passive and not resist or collaborate in the movement. Measurements were performed twice, and the protocol was repeated after 10 minutes of recovery. Resting and peak blood flow during PLM were calculated; the area under the blood flow versus time curve (AUC) was also derived by calculating the integral of the function over 60 seconds, after subtracting the basal value at rest.^9^

### Statistical analysis

Quantitative variables were summarized as mean and SD. For the comparison of continuous variables, the independent *t*‐test was used for variables with normal distribution and homogeneous variances, and the Mann–Whitney *U* test was used for nonparametric distributions. Categorical variables were compared using Pearson's Chi‐square test; if the expected frequencies in a cell were less than five, Fisher's exact test was used to ensure better statistical accuracy. A *P* value <0.05 was considered statistically significant. All statistical analyses were performed using SPSS version 15 software.

## RESULTS

### Participants

A total of 21 patients with SSc (19 women, 90%) and 21 age‐ and sex‐matched healthy controls were included in the study (Table 1). No differences were observed between the SSc group and controls in terms of body mass, height, body mass index, and body composition (see Table 1). Twelve participants in each group reported a sedentary lifestyle, whereas the remaining individuals engaged in light physical activity (eg, walking, gym, and cycling) only once a week.

**Table 1:** Characteristics of patients with SSc and controls*

	Patients (SSc)	Controls
n	21	21
Female, n (%)	19 (90)	18 (86)
Age, y	69.3 (±8.53)	66.4 (±4.91)
Height, m	1.62 (±0.06)	1.65 (±0.07)
Weight, kg	61.8 (±9.55)	68.3 (±12.8)
BMI	23.63 (±3.50)	24.95 (±3.92)
FM, %	23.6 (±8.2)	27.2 (±7.5)
FFM, %	76.4 (±8.2)	72.8 (±7.6)
ALM, kg	16.9 (±2.6)	18.2 (±3.4)

* Values are reported as mean (±SD) unless otherwise indicated. ALM, appendicular lean mass; BMI, body mass index; FM, fat mass; FFM, fat‐free mass; SSc, systemic sclerosis.

The average disease duration in the SSc group was 16.2 ± 9.8 years. The lcSSc was the most prevalent phenotype, observed in 15 patients (71%). All patients tested positive for antinuclear antibodies, with anticentromere antibodies being the most common (47%), followed by anti‐Scl70 antibodies (43%). Nine patients (43%) had a history of digital ulcers (DU), and 8 (38%) were diagnosed with PAH via right heart catheterization. Additionally, 11 patients (52%) presented with interstitial lung disease (ILD) (Table 2). In terms of pharmacological treatments and comorbidities, 17 patients (81%) were receiving vasoactive therapies, primarily for DU or PAH. Among these, nine patients were taking intravenous prostacyclin, with a mean interval of 11 ± 6 days between the last infusion and the PLM assessment. Six patients with DU and four with PAH were undergoing dual combination therapy, whereas one patient was receiving triple therapy. Eight patients were receiving immunosuppressive treatment. All patients with SSc were under treatment for cardiovascular conditions, including antihypertensives, statins, anticoagulants, or antiplatelet agents (Table 3). A history of major cardiovascular events was reported in eight patients, and six had a history of cancer. In the control group, 17 of 21 patients were taking antihypertensives, and 6 were receiving statin therapy; 2 had a history of solid tumors. No significant differences were found between patients and controls regarding statin use (*P* = 0.74) or cancer history (*P* = 0.24). However, cardiovascular events were significantly more frequent in the SSc group (*P* = 0.03), which was further supported by a significantly higher Charlson Comorbidity Index (CCI) in patients compared to controls (*P* < 0.0001).

**Table 2:** Phenotype of patients with SSc*

	SSc
Disease duration, y	16.2 (±9.78)
SSc phenotype, n (%)	
lcSSc	15 (71)
dcSSc	6 (29)
mRSS	9 (±6.67)
Antibodies, n (%)	
Anticentromere	10 (47)
Anti‐Scl70	9 (43)
Anti–RNA polymerase III	1 (5)
Anti–SSA/Ro52	1 (5)
DU, n (%)	9 (43)
PAH, n (%)	8 (38)
ILD, n (%)	11 (52)
CVD, n (%)	8 (38)
Tumors, n (%)	6 (28)

* Values are reported as mean (±SD) unless otherwise indicated. CVD, cardiovascular disease; dcSSc, diffuse cutaneous SSc; DU, digital ulcers; ILD, interstitial lung disease; lcSSc, limited cutaneous SSc; mRSS, modified Rodnan Skin Score; PAH, pulmonary arterial hypertension; SSc, systemic sclerosis.

**Table 3:** SSc therapies*

Therapies	n (%)
Current immunosuppression	9 (43)
Hydroxychloroquine	1 (11)
Mycophenolate mofetil	2 (22)
Azathioprine	1 (11)
Tacrolimus	1 (11)
Rituximab	2 (22)
Tocilizumab	2 (22)
Vasoactive drugs	17 (81)
PCA	9 (53)
ERA	13 (76)
PDE5i	4 (23)
Riociguat	2 (12)
Pentoxifylline	1 (6)
Dual therapy	10 (59)
PCA + ERA	6 (60)
ERA + PDE5i	2 (20)
ERA + riociguat	2 (20)
Other therapies	
Nintedanib	2 (9)
Statins	7 (33)

* ERA, endothelin receptor antagonist; PCA, prostacyclin analog; PDE5i, phosphodiesterase 5 inhibitor; SSc, systemic sclerosis.

### PLM response: SSc versus controls

**Figure 1:**
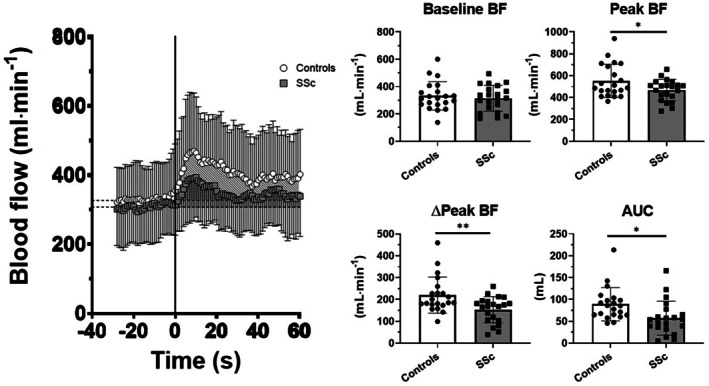
PLM results. Values are reported as mean ± SD. *P < 0.05; **P < 0.01. AUC, area under the curve; BF, blood flow; ∆Peak, changes from baseline to peak blood flow; PLM, passive leg movement; SSc, systemic sclerosis.

At baseline, no significant differences were observed between the groups in terms of CFA diameter (*P* = 0.14) or blood flow (*P* = 0.56) (see Figure 1). Blood flow increased with PLM in both groups; however, a significantly blunted hyperemic response was observed in patients with SSc compared to controls. Specifically, peak blood flow was lower in patients versus controls (*P* = 0.038), as was the change from baseline (ΔPeak) (*P* = 0.005) and the AUC (*P* = 0.01) (Table 4, Figure 1).

**Table 4:** PLM results*

	Diameter CFA, mm	Baseline BF, mL/min	Peak BF, mL/min	ΔPeak BF, mL/min	AUC, mL
Patients					
Mean	8.0	314.4	467.7	153.3	57.1
SD	0.9	94.5	97.8	59.8	38.9
Controls					
Mean	8.4	332.3	552.5	220.2	89.3
SD	0.9	104.1	151.9	82.7	38.1
*P* value	0.140	0.562	0.038	0.005	0.010

* AUC, area under the curve; BF, blood flow; CFA, common femoral artery.

### PLM results and SSc clinical phenotypes

Further analyses explored the influence of clinical phenotype and pharmacologic treatment on PLM parameters. No significant differences were observed between patients with lcSSc and those with dcSSc regarding peak blood flow (*P* = 0.34), ΔPeak (*P* = 0.57), or AUC (*P* = 0.30). Disease duration (*P* = 0.97) and comorbidity burden (*P* = 0.52) did not affect PLM responses. Similarly, anti‐Scl70 antibody positivity did not correlate with PLM outcomes (peak: *P* = 0.13; ΔPeak: *P* = 0.97; AUC: *P* = 0.70). However, a significant inverse correlation was found between the modified Rodnan Skin Score (mRSS) and CFA diameter, with higher mRSS associated with a smaller artery diameter (7.81 vs 8.14 mm; *P* = 0.001).

Patients with a history of DU showed no significant differences in PLM parameters compared to those without DU (peak: *P* = 1; ΔPeak: *P* = 1; AUC: *P* = 0.65) (see Table 5). Similarly, PAH did not significantly influence the PLM response, although a trend toward higher peak blood flow was observed in patients with PAH compared to those without (*P* = 0.05). The presence of ILD was not associated with significant differences in PLM parameters (peak: *P* = 0.65; ΔPeak: *P* = 0.28; AUC: *P* = 0.60) (Table 5).

**Table 5:** PLM and SSc manifestations*

	Peak BF, mL/min	ΔPeak BF, mL/min	AUC, mL
DU	478.4 (±112.7)	153.4 (±61.8)	49.5 (±29.6)
Not DU	459.6 (±89.3)	153.2 (±61)	62.8 (±45.1)
*P* value	1	1	0.65
PAH	516.4 (±53.9)	154.6 (±59.8)	55.3 (±38.6)
Not PAH	437.7 (±108.1)	152.5 (±62.2)	58.2 (±40.7)
*P* value	0.05	0.75	0.97
ILD	460.7 (±100.4)	167.7 (±60.9)	55.1 (±41.1)
Not ILD	475.3 (±99.6)	137.4 (±57.4)	59.3 (±38.4)
*P* value	0.65	0.28	0.60

* Values are reported as mean (±SD). AUC, area under the curve; BF, blood flow; DU, digital ulcers; ILD, interstitial lung disease; PAH, pulmonary arterial hypertension; PLM, passive leg movement; SSc, systemic sclerosis.

Among the eight patients with cardiovascular events, the most frequent manifestations were venous thrombosis (37.5%), followed by stroke (25%) and arterial stenosis (25%). Two patients had non‐ST‐elevation myocardial infarction (NSTEMI) and pulmonary embolism (PE), respectively. In this subgroup, the median peak was 467.6 (interquartile range [IQR] 352–518) compared with 501 (IQR 431–512) in patients without cardiovascular history. The median AUC was 41.9 (IQR 38.5–67.6) and 57.3 (IQR 19.7–65.6), respectively. Patients with NSTEMI and PE and one patient with venous thrombosis had N‐terminal pro–B‐type natriuretic peptide (NTproBNP) values >300 ng/L. Overall, six patients had NTproBNP values >300 ng/L; four of six had PAH, and three of these were receiving dual vasoactive therapy. C‐reactive protein (CRP) levels were within the normal range (<5 mg/L) in all but one patient due to a recent infection. No cardiovascular sequela was observed; the examination was well tolerated and no EKG abnormalities occurred during PLM.

### PLM results and treatments

No differences in PLM response were noted between patients receiving immunosuppressants and those without background therapy (peak: *P* = 0.8; ΔPeak: *P* = 0.54; AUC: *P* = 0.97), nor between those receiving vasoactive therapies and untreated patients (peak: *P* = 0.96; ΔPeak: *P* = 0.52; AUC: *P* = 0.17). Similarly, no significant differences were found in patients receiving NO‐mediated or antiendothelin therapies (peak: *P* = 0.62 and 0.41; ΔPeak: *P* = 0.97 and 0.97; AUC: *P* = 0.91 and 0.86, respectively). Interestingly, patients taking statins exhibited significantly lower baseline blood flow compared to those not receiving statin therapy (252.1 vs 345.5 mL/min; *P* = 0.031). Finally, neither a history of cardiovascular events nor previous cancer significantly influenced PLM parameters within the SSc group.

## DISCUSSION

Endothelial dysfunction is a central mechanism in the pathogenesis of SSc, influencing disease manifestations and significantly impacting prognosis.^2^ Among the techniques available for assessing endothelial health, FMD has been the most widely used to evaluate conduit artery responses to NO‐mediated vasodilation.^17^ Our study is the first to employ PLM to assess endothelial dysfunction in SSc while stratifying patients by disease phenotype, presence of comorbidities, and therapeutic regimens. Consistent with previous findings,^11^ patients with SSc demonstrated significantly impaired PLM responses compared to healthy controls across multiple parameters, including peak blood flow, changes in blood flow from baseline (∆Peak), and area under the curve (AUC). These results reinforce the hypothesis that reduced NO bioavailability, due to alterations in endothelial NO synthase and inducible NO synthase balance, underlies the vascular impairment observed in SSc.^18^, ^19^, ^20^ The higher prevalence of cardiovascular events and elevated CCI in our patient cohort further supports the systemic impact of vascular dysfunction in SSc and underscores the prognostic value of comorbid conditions. These findings align with the broader literature, which recognizes vascular complications, particularly PAH and DU, as major contributors to morbidity and mortality in this patient population.^21^, ^22^

Interestingly, no significant differences in PLM response were found between lcSSc and dcSSc disease phenotypes or according to autoantibody profiles. This uniformity suggests that endothelial dysfunction, as measured by PLM, may be a common pathophysiologic feature in SSc independent of clinical or serological subtype. Additionally, an inverse relationship between the mRSS and CFA diameter was observed, supporting the link between dermal fibrosis and vascular remodeling. This relationship may reflect the process of endothelial‐to‐mesenchymal transition, which contributes to intimal hyperplasia, luminal narrowing, and vessel wall stiffness.

The findings from PLM were juxtaposed with existing literature on FMD. Studies such as those by Frech et al^23^ and Takahashi et al^24^ have demonstrated diminished FMD responses in lcSSc, particularly in patients with DU and elevated pulmonary arterial pressure, highlighting the functional consequences of NO deficiency. However, our PLM data did not reveal significant differences between patients with SSc with and without a history of DU.^12^, ^23^, ^24^ This discrepancy could be attributed to several factors, including the small number of DU‐positive patients in our cohort and the fact that all such patients were receiving continuous vasoactive therapy, which may have masked the degree of vascular dysfunction. This differs from other studies in which vasoactive medications were suspended before testing.^12^, ^25^

Among patients with PAH, no overall reduction in PLM response was observed. Interestingly, this subgroup demonstrated a slightly higher peak blood flow compared to non‐PAH patients. Although not statistically conclusive, this trend may reflect the beneficial effects of early and aggressive treatment regimens on peripheral vascular reactivity. It is notable that these pharmacologic agents enhance vasodilation at the level of arterioles and microcirculation, which may contribute to the augmented hyperemic response observed. Furthermore, peripheral vasodilation has been shown to have a pivotal role in PLM‐induced hyperemia compared to cardiac output, whose increase has not been associated with an increased perfusion pressure, which tends to decrease.^9^

Similarly, the presence of ILD did not correlate with altered PLM responses. This is consistent with the nature of PLM as a peripheral vascular assessment tool, which may not capture parenchymal pulmonary pathology such as inflammation or fibrosis. These findings reinforce the specific utility of PLM in evaluating systemic and peripheral endothelial function rather than organ‐specific involvement.

With regard to therapeutic interventions, our study found no significant association between PLM responses and the use of immunosuppressive or vasoactive agents.

These results align with current clinical understanding that although such treatments can alleviate symptoms and delay disease progression, they do not reverse endothelial injury or restore vascular homeostasis. Microvascular chronic damage and impaired shear‐stress–induced NO release may reduce the capacity of vasoactive therapies to augment vasodilation during PLM. It is worth highlighting that some studies using FMD in patients with SSc have reported encouraging data on the role of vasoactive therapies, such as bosentan, in improving vascular response.^23^, ^25^ However, further studies are needed to better stratify patients according to disease duration, severity, and treatment duration and to compare the two techniques. Patients receiving immunosuppressors had normal CRP levels like patients not receiving treatment, except for two patients with a recent infection. Clifton et al reported higher CRP values in patients with SSc compared with healthy controls; however, CRP levels were not predictive of PLM response, and only 17% of patients in their cohort were taking an immunosuppressor.^12^ In our study, CRP values from healthy controls were not available, but the overall normal CRP levels observed in patients with SSc likely reflect the ability of immunosuppressive therapies to modulate systemic inflammation. Normal CRP may not necessarily exclude persistent microvascular dysfunction, which may occur independently of systemic inflammatory activity. Importantly, PAH remains a leading cause of mortality in SSc, and despite improvements in survival and quality of life afforded by current treatments, no existing therapy directly targets the primary endothelial dysfunction central to disease pathogenesis.^21^, ^22^

A noteworthy finding in our study was the significantly lower baseline blood flow observed in patients treated with statins. Although this does not establish causality, the result is consistent with the pleiotropic effects of statins on endothelial biology and modulation of immune responses.^26^, ^27^, ^28^ Preclinical and clinical studies have further demonstrated that statins enhance NO production, reduce levels of endothelin‐1 and VEGF, and improve FMD outcomes.^29^, ^30^, ^31^, ^32^ However, despite these promising findings, the potential disease‐modifying role of statins in SSc remains speculative and warrants further investigation through longitudinal studies.

Methodologically, PLM offers several advantages over FMD. Unlike FMD, PLM does not require precise imaging of vessel diameter, as the CFA remains relatively unchanged during exercise.^17^ Moreover, PLM predominantly reflects microvascular function and the capacity for arteriolar dilation, which may be more relevant in diseases with prominent microcirculatory pathology. PLM is also highly dependent on NO bioavailability, with NO synthase inhibition shown to reduce vascular responses by up to 90%. In contrast, FMD's dependency on NO is less consistent across studies.^33^, ^34^, ^35^, ^36^ Although both techniques are valuable and complementary, the distinct mechanisms they assess suggest that PLM may provide additional insight in settings where microvascular integrity is compromised.

Previous studies, including those by Rossman et al, have demonstrated moderate correlations between PLM and FMD responses when controlling for shear stress. However, these correlations weaken when adjustments are made for flow stimuli, indicating that the two modalities assess distinct aspects of vascular physiology.^17^ In the context of SSc, this differentiation is particularly relevant, as both structural and functional microvascular abnormalities are hallmark features of the disease.

Despite the strengths of our study, including the novel application of PLM and detailed phenotypic stratification, several limitations must be acknowledged. The small sample size and predominance of patients with longstanding disease may have limited our ability to detect subtle differences across subgroups. Additionally, ongoing therapies, especially vasoactive agents, may have influenced vascular responses, despite efforts to standardize assessments. Of note, a major limitation of our study is the absence of circulating biomarkers, such as NO metabolites, endothelin‐1, or inflammatory cytokines, which could have provided mechanistic insights and strengthened the interpretation of PLM responses. Our findings should thus be interpreted with caution and considered hypothesis‐generating rather than definitive.

Finally, the partial attenuation of PLM responses following administration of NO synthase inhibitors, as observed in other studies, suggests that additional mediators may contribute to the hyperemic response.^33^, ^34^, ^36^, ^37^ This complexity further underscores the need for integrative approaches to understanding and treating vascular dysfunction in SSc.

To conclude, PLM is a practical, noninvasive method to assess NO‐mediated endothelial dysfunction in SSc. Its simplicity, reproducibility, and focus on microvascular function make it suitable for early disease monitoring and for evaluating future targeted therapies. The lack of correlation with current treatments highlights their limited effect on vascular injury and the need for strategies that directly improve endothelial health. Larger, longitudinal studies are needed to confirm PLM's clinical and research utility.

## AUTHOR CONTRIBUTIONS

All authors contributed to at least one of the following manuscript preparation roles: conceptualization AND/OR methodology, software, investigation, formal analysis, data curation, visualization, and validation AND drafting or reviewing/editing the final draft. As corresponding author, Dr Quartuccio confirms that all authors have provided the final approval of the version to be published and takes responsibility for the affirmations regarding article submission (eg, not under consideration by another journal), the integrity of the data presented, and the statements regarding compliance with institutional review board/Declaration of Helsinki requirements.

## Supporting information


**Disclosure Form**:

## References

[1] Pope JE , Denton CP , Johnson SR , et al. State‐of‐the‐art evidence in the treatment of systemic sclerosis. Nat Rev Rheumatol 2023;19:212–226. 36849541 10.1038/s41584-023-00909-5 PMC9970138

[2] Patnaik E , Lyons M , Tran K , et al. Endothelial dysfunction in systemic sclerosis. Int J Mol Sci 2023;24:14385. 37762689 10.3390/ijms241814385 PMC10531630

[3] Abraham D , Distler O . How does endothelial cell injury start? The role of endothelin in systemic sclerosis. Arthritis Res Ther 2007;9(suppl 2):S2. 10.1186/ar2186 PMC2072886 17767740

[4] Rosendahl A‐H , Schönborn K , Krieg T . Pathophysiology of systemic sclerosis (scleroderma). Kaohsiung J Med Sci 2022;38:187–195. 35234358 10.1002/kjm2.12505 PMC11896191

[5] Mihai C , Landewé R , van der Heijde D , et al; EUSTAR co‐authors . Digital ulcers predict a worse disease course in patients with systemic sclerosis. Ann Rheum Dis 2016;75:681–686. 25688073 10.1136/annrheumdis-2014-205897

[6] Carrai V , Miniati I , Guiducci S , et al. Evidence for reduced angiogenesis in bone marrow in SSc: immunohistochemistry and multiparametric computerized imaging analysis. Rheumatology (Oxford) 2012;51:1042–1048. 22271757 10.1093/rheumatology/ker447

[7] Raschi E , Privitera D , Bodio C , et al. Scleroderma‐specific autoantibodies embedded in immune complexes mediate endothelial damage: an early event in the pathogenesis of systemic sclerosis. Arthritis Res Ther 2020;22:265. 33168071 10.1186/s13075-020-02360-3 PMC7654597

[8] Harris RA , Nishiyama SK , Wray DW , et al. Ultrasound assessment of flow‐mediated dilation. Hypertension 2010;55:1075–1085. 20351340 10.1161/HYPERTENSIONAHA.110.150821 PMC2878744

[9] Gifford JR , Richardson RS . CORP: ultrasound assessment of vascular function with the passive leg movement technique. J Appl Physiol (1985) 2017;123:1708–1720. 10.1152/japplphysiol.00557.2017 PMC5814681 28883048

[10] Di Minno MN , Ambrosino P , Lupoli R , et al. Clinical assessment of endothelial function in patients with rheumatoid arthritis: a meta‐analysis of literature studies. Eur J Intern Med 2015;26:835–842. 26547241 10.1016/j.ejim.2015.10.016

[11] Moroni L , Selmi C , Angelini C , et al. Evaluation of endothelial function by flow‐mediated dilation: a comprehensive review in rheumatic disease. Arch Immunol Ther Exp (Warsz) 2017;65:463–475. 28361180 10.1007/s00005-017-0465-7

[12] Clifton HL , Machin DR , Groot HJ , et al. Attenuated nitric oxide bioavailability in systemic sclerosis: evidence from the novel assessment of passive leg movement. Exp Physiol 2018;103:1412–1424. 29790215 10.1113/EP086991 PMC6167160

[13] Ives SJ , Layec G , Hart CR , et al. Passive leg movement in chronic obstructive pulmonary disease: evidence of locomotor muscle vascular dysfunction. J Appl Physiol (1985) 2020;128:1402–1411. 10.1152/japplphysiol.00568.2019 PMC7272759 32324478

[14] Nelson AD , Rossman MJ , Witman MA , et al. Nitric oxide‐mediated vascular function in sepsis using passive leg movement as a novel assessment: a cross‐sectional study. J Appl Physiol (1985) 2016;120:991–999. 10.1152/japplphysiol.00961.2015 PMC6345212 26869709

[15] Zuccarelli L , Baldassarre G , Magnesa B , et al. Peripheral impairments of oxidative metabolism after a 10‐day bed rest are upstream of mitochondrial respiration. J Physiol 2021;599:4813–4829. 34505290 10.1113/JP281800 PMC9293208

[16] van den Hoogen F , Khanna D , Fransen J , et al. 2013 classification criteria for systemic sclerosis: an American College of Rheumatology/European League Against Rheumatism collaborative initiative. Arthritis Rheum 2013;65(11):2737–2747. 24122180 10.1002/art.38098 PMC3930146

[17] Rossman MJ , Groot HJ , Garten RS , et al. Vascular function assessed by passive leg movement and flow‐mediated dilation: initial evidence of construct validity. Am J Physiol Heart Circ Physiol 2016;311:H1277–H1286. 27638879 10.1152/ajpheart.00421.2016 PMC5130489

[18] Cotton SA , Herrick AL , Jayson MI , et al. Endothelial expression of nitric oxide synthases and nitrotyrosine in systemic sclerosis skin. J Pathol 1999;189:273–278. 10547586 10.1002/(SICI)1096-9896(199910)189:2<273::AID-PATH413>3.0.CO;2-4

[19] Luo JY , Liu X , Jiang M , et al. Oxidative stress markers in blood in systemic sclerosis: a meta‐analysis. Mod Rheumatol 2017;27:306–314. 27425641 10.1080/14397595.2016.1206510

[20] Allanore Y , Borderie D , Hilliquin P , et al. Low levels of nitric oxide (NO) in systemic sclerosis: inducible NO synthase production is decreased in cultured peripheral blood monocyte/macrophage cells. Rheumatology (Oxford) 2001;40:1089–1096. 11600736 10.1093/rheumatology/40.10.1089

[21] Morrisroe K , Stevens W , Huq M , et al; Australian Scleroderma Interest Group (ASIG) . Survival and quality of life in incident systemic sclerosis‐related pulmonary arterial hypertension. Arthritis Res Ther 2017;19:122. 28576149 10.1186/s13075-017-1341-x PMC5457656

[22] Naranjo M , Hassoun PM . Systemic sclerosis‐associated pulmonary hypertension: spectrum and impact. Diagnostics (Basel) 2021;11:911. 34065226 10.3390/diagnostics11050911 PMC8161029

[23] Frech T , Walker AE , Barrett‐O'Keefe Z , et al. Systemic sclerosis induces pronounced peripheral vascular dysfunction characterized by blunted peripheral vasoreactivity and endothelial dysfunction. Clin Rheumatol 2015;34:905–913. 25511849 10.1007/s10067-014-2834-5 PMC4757465

[24] Takahashi T , Asano Y , Amiya E , et al. Clinical correlation of brachial artery flow‐mediated dilation in patients with systemic sclerosis. Mod Rheumatol 2014;24:106–111. 24261766 10.3109/14397595.2013.854064

[25] Sfikakis PP , Papamichael C , Stamatelopoulos KS , et al. Improvement of vascular endothelial function using the oral endothelin receptor antagonist bosentan in patients with systemic sclerosis. Arthritis Rheum 2007;56:1985–1993. 17530638 10.1002/art.22634

[26] Greenwood J , Steinman L , Zamvil SS . Statin therapy and autoimmune disease: from protein prenylation to immunomodulation. Nat Rev Immunol 2006; 6:358–370. 16639429 10.1038/nri1839 PMC3842637

[27] Zhang X , Jin J , Peng X , et al. Simvastatin inhibits IL‐17 secretion by targeting multiple IL‐17‐regulatory cytokines and by inhibiting the expression of IL‐17 transcription factor RORC in CD4+ lymphocytes. J Immunol 2008;180:6988–6996. 18453621 10.4049/jimmunol.180.10.6988

[28] Khattri S , Zandman‐Goddard G . Statins and autoimmunity. Immunol Res 2013;56:348–357. 23572428 10.1007/s12026-013-8409-8

[29] Ladak K , Pope JE . A review of the effects of statins in systemic sclerosis. Semin Arthritis Rheum 2016;45:698–705. 26639033 10.1016/j.semarthrit.2015.10.013

[30] Del Papa N , Cortiana M , Vitali C , et al. Simvastatin reduces endothelial activation and damage but is partially ineffective in inducing endothelial repair in systemic sclerosis. J Rheumatol 2008;35:1323–1328. 18528965

[31] Rossi M , Bazzichi L , Ghiadoni L , et al. Increased finger skin vasoreactivity and stimulated vasomotion associated with simvastatin therapy in systemic sclerosis hypercholesterolemic patients. Rheumatol Int 2012;32:3715–3721. 22057138 10.1007/s00296-011-2183-5

[32] Abou‐Raya A , Abou‐Raya S , Helmii, M . Statins: potentially useful in therapy of systemic sclerosis‐related Raynaud's phenomenon and digital ulcers. J Rheumatol 2008;35:1801–1808. 18709692

[33] Mortensen SP , Askew CD , Walker M , et al. The hyperaemic response to passive leg movement is dependent on nitric oxide: a new tool to evaluate endothelial nitric oxide function. J Physiol 2012;590:4391–4400. 22733658 10.1113/jphysiol.2012.235952 PMC3473293

[34] Trinity JD , Groot HJ , Layec G , et al. Nitric oxide and passive limb movement: a new approach to assess vascular function. J Physiol 2012;590:1413–1425. 22310310 10.1113/jphysiol.2011.224741 PMC3382331

[35] Green, D. Point : Flow‐mediated dilation does reflect nitric oxide‐mediated endothelial function. J Appl Physiol (1985) 2005;99:1233–1234; discussion 1237–1238. 10.1152/japplphysiol.00601.2005 16103524

[36] Wray DW , Witman MA , Ives SJ , et al. Does brachial artery flow‐mediated vasodilation provide a bioassay for NO? Hypertension 2013;62:345–351. 23774225 10.1161/HYPERTENSIONAHA.113.01578 PMC3775568

[37] Groot HJ , Trinity JD , Layec G , et al. The role of nitric oxide in passive leg movement‐induced vasodilatation with age: insight from alterations in femoral perfusion pressure. J Physiol 2015;593:3917–3928. 26108562 10.1113/JP270195 PMC4575577

